# Upgrade of crystallography beamline BL19U1 at the Shanghai Synchrotron Radiation Facility

**DOI:** 10.1107/S1600576724002188

**Published:** 2024-04-15

**Authors:** Qingjie Xiao, Tingting Wu, Kangwen Bao, Jianchao Tang, Yupu Zhang, Weizhe Zhang, Zhongjie Zhu, Yijun Gu, Shuyu Zhou, Chunyu Li, Lanlu Lu, Luozhen Jiang, Yang Liu, Wenming Qin

**Affiliations:** a National Facility for Protein Science in Shanghai, Shanghai Advanced Research Institute, Chinese Academy of Sciences, Shanghai, 201210, People’s Republic of China; HPSTAR and Harbin Institute of Technology, People’s Republic of China

**Keywords:** crystallography beamline BL19U1, upgrades, Shanghai Synchrotron Radiation Facility

## Abstract

BL19U1, an energy-tunable protein complex crystallography beamline at the Shanghai Synchrotron Radiation Facility, has emerged as one of the most productive MX beamlines since opening to the public in July 2015. As of October 2023, it has contributed to over 2000 protein structures deposited in the Protein Data Bank (PDB), resulting in the publication of more than 1000 scientific papers. In response to increasing interest in structure-based drug design utilizing X-ray crystallography for fragment library screening, enhancements have been implemented in both hardware and data collection systems on the beamline to optimize efficiency. Hardware upgrades include the transition from MD2 to MD2S for the diffractometer, alongside the installation of a humidity controller featuring a rapid nozzle exchanger. This allows users to opt for either low-temperature or room-temperature data collection modes. The control system has been upgraded from *Blu-Ice* to *MXCuBE3*, which supports website-mode data collection, providing enhanced compatibility and easy expansion with new features. An automated data processing pipeline has also been developed to offer users real-time feedback on data quality.

## Abstract 2

The protein complex crystallography beamline BL19U1 is preparing for the establishment of an automated drug screening platform by upgrading both hardware and data collection control systems to optimize efficiency. The upgrade of the experimental station devices and data collection control system on the BL19U1 beamline are reported in this paper.

## 1. Introduction

Numerous physiological processes in living organisms, including growth, development, ageing and disease formation, involve biological macromolecules such as proteins and nucleic acids. These macromolecules play a pivotal role in determining various life functions and activities. Given that molecular function is intricately tied to structure, unveiling the three-dimensional structure of biological macromolecules becomes paramount for understanding the fundamental mechanisms underlying these processes (Watson *et al.*, 2005 ▸). Delving into the three-dimensional structures of these mol­ecules allows for a more profound exploration of their roles in biological processes, providing robust support for the development of structure-based drugs.

X-ray crystallography has been indispensable in elucidating the structures of biological macromolecules. The BL19U1 beamline, situated at the third-generation Shanghai Synchrotron Radiation Facility (SSRF) in China, stands out as a macromolecular crystallography (MX) beamline specifically designed for proteins with substantial molecular weight and crystals with large unit-cell dimensions. The BL19U1 beamline boasts features such as high-throughput photons, a low-divergence incident X-ray beam and a large effective area detector (Zhang *et al.*, 2019 ▸; Jiang *et al.*, 2009 ▸). The beam intensity is 1.5 × 10^12^ photons per second at 12 keV (at 300 mA) and the focused beam spot size is 80 × 40 µm (horizontal × vertical). Since its inception in 2015, over 2000 crystal structures with diffraction data collected on BL19U1 have been deposited in the Protein Data Bank (PDB, https://www.rcsb.org/), establishing it as one of the most productive MX beamlines in the world.

**Figure 1:**
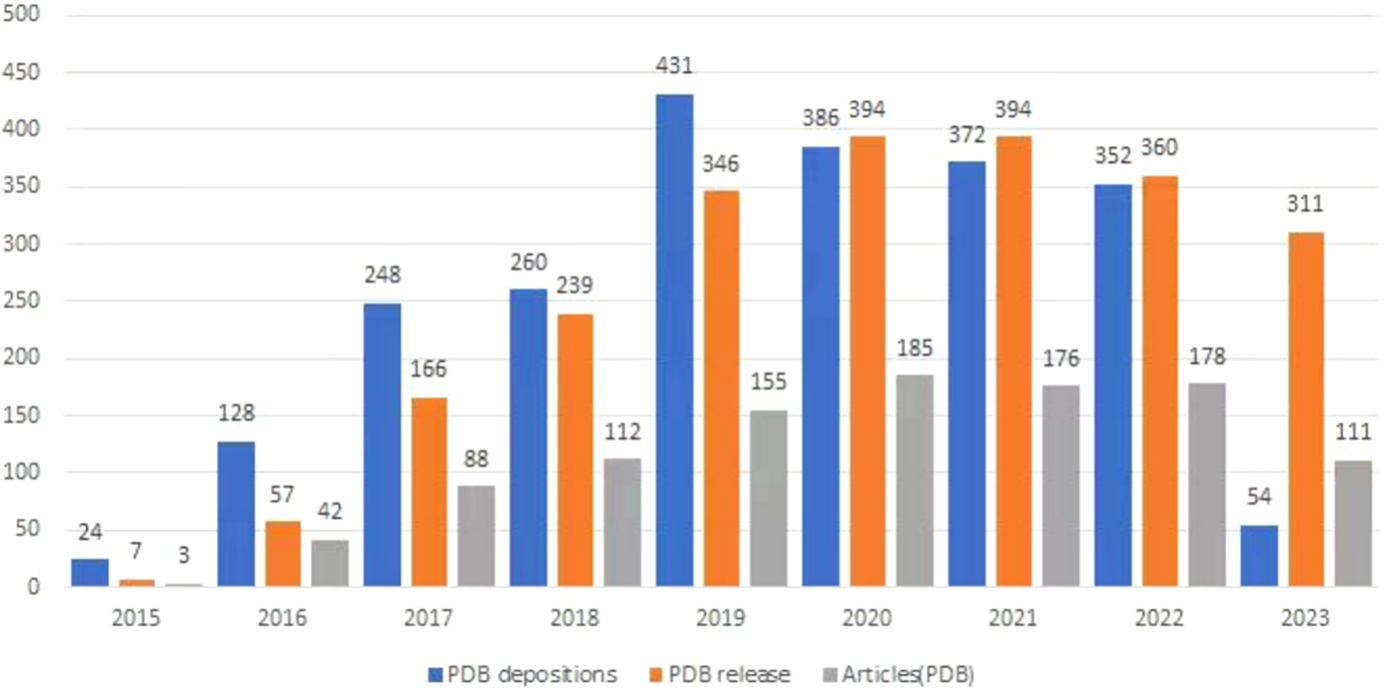
Statistical analysis related to the number of structure depositions, the release of structures and the number of articles. PDB data are derived from the BioSync website (https://biosync.sbkb.org).

Fig. 1 ▸, derived from the BioSync website (https://biosync.sbkb.org; Kuller *et al.*, 2002 ▸), visually represents data tracking the number of PDB publications and related articles up to 11 October 2023. The analysis reveals notable patterns: a substantial increase in PDB deposition, from 24 in 2015 to 431 in 2019, and, despite a decrease post-2019, a consistently high number with 352 structures submitted in 2022. Similarly, released PDB crystal structures increased annually from seven in 2015 to 394 in 2020, with a slight decrease to 360 in 2022. The parallel increase in published articles, peaking at 185 and maintaining high levels subsequently, underscores BL19U1’s role in advancing scientific research, with consistently high levels of PDB data releases and publications.

With the ongoing advances in scientific research and technology, an escalating number of researchers and businesses are expressing a keen interest in fragment library screening based on X-ray crystallography methods. This method not only proves effective in identifying molecules with low affinity but also serves as a valuable tool for thoroughly exploring numerous potential binding sites, thus facilitating the development of promising new drugs. Over the past few years, several automatic fragment screening MX beamlines, including I-104 at Diamond Light Source (UK) and the BioMAX beamline at MAXIV (Sweden), have been established (Douangamath *et al.*, 2021 ▸; Lima *et al.*, 2020 ▸; Ursby *et al.*, 2020 ▸). In particular, the Diamond I-104 beamline has established a mature fragmentation screening platform, successfully screening fragments for many important drug target proteins with a 1% to 30% hit rate (Douangamath *et al.*, 2021 ▸, 2020 ▸; Newman *et al.*, 2021 ▸). To establish similar platforms, enhancements to the stability and quality of BL19U1’s hardware have been implemented. Simultaneously, the automation level of the beamline, covering sample transfer, sample centring, data collection, data processing and optimization of structural analysis technology, needs improvement to ensure the efficiency of crystal drug screening.

To meet the evolving requirements and challenges in this scientific research field, the hardware equipment and software system of BL19U1 have undergone updates. This includes the replacement of the diffractometer with an updated version in July 2023, the establishment of a new data collection control system in September 2023, the installation of a humidity controller (HC) in July 2020 and the development of an automated diffraction data processing pipeline in August 2021. Future plans involve upgrading the sample-changer system to improve sample loading efficiency and increase sample storage capacity.

## 2. Experimental station

The protein complex crystallography beamline BL19U1 comprises the undulator source, front end, optical elements and an experimental station, constituting a sophisticated setup for advanced crystallography studies. In contrast to a conventional laboratory source X-ray diffractometer, where X-rays are generated using copper or molybdenum targets, BL19U1 derives its X-rays from the SSRF synchrotron radiation source, resulting in significantly higher light intensity spanning several orders of magnitude, with the added advantage of tunable wavelengths (Zhang *et al.*, 2019 ▸; Jiang *et al.*, 2009 ▸). These characteristics enhance the precision and versatility of experiments conducted on the beamline.

The exceptional beam intensity of the SSRF, coupled with the utilization of a PILATUS detector capable of rapidly reading crystal diffraction signals, enables the acquisition of a single frame of diffraction from a crystal within a few hundred milliseconds or even less. This remarkable speed significantly amplifies data collection efficiency, allowing researchers to obtain high-quality data swiftly.

Moving down the crystallography beamline, the experimental station, situated at the beamline’s terminus, houses critical equipment pivotal to data collection. This includes detectors, diffractometers, sample changers, fluorescence detectors, cold streams, ion chambers, attenuators and more, all intricately linked to the data acquisition process. Recent years have witnessed substantial upgrades to key equipment within the experimental station, specifically aimed at enhancing data collection efficiency, especially in the context of fragment library screening with crystals.

One noteworthy upgrade involves the replacement and enhancement of the macrodiffractometer MD2 with its upgraded version MD2S (https://www.arinax.com/md2-s-x-ray-microdiffractometer/). Other notable changes include the separation of the capillary and beamstop, with the added benefit of a movable beamstop position. This modification significantly expands the available space for sample placement, particularly accommodating *in situ* data collection with plate screens. *In situ* diffraction using crystal plates holds immense promise for crystal-based fragment library screening, effectively circumventing issues associated with the freezing and capture of crystals.

**Figure 2:**
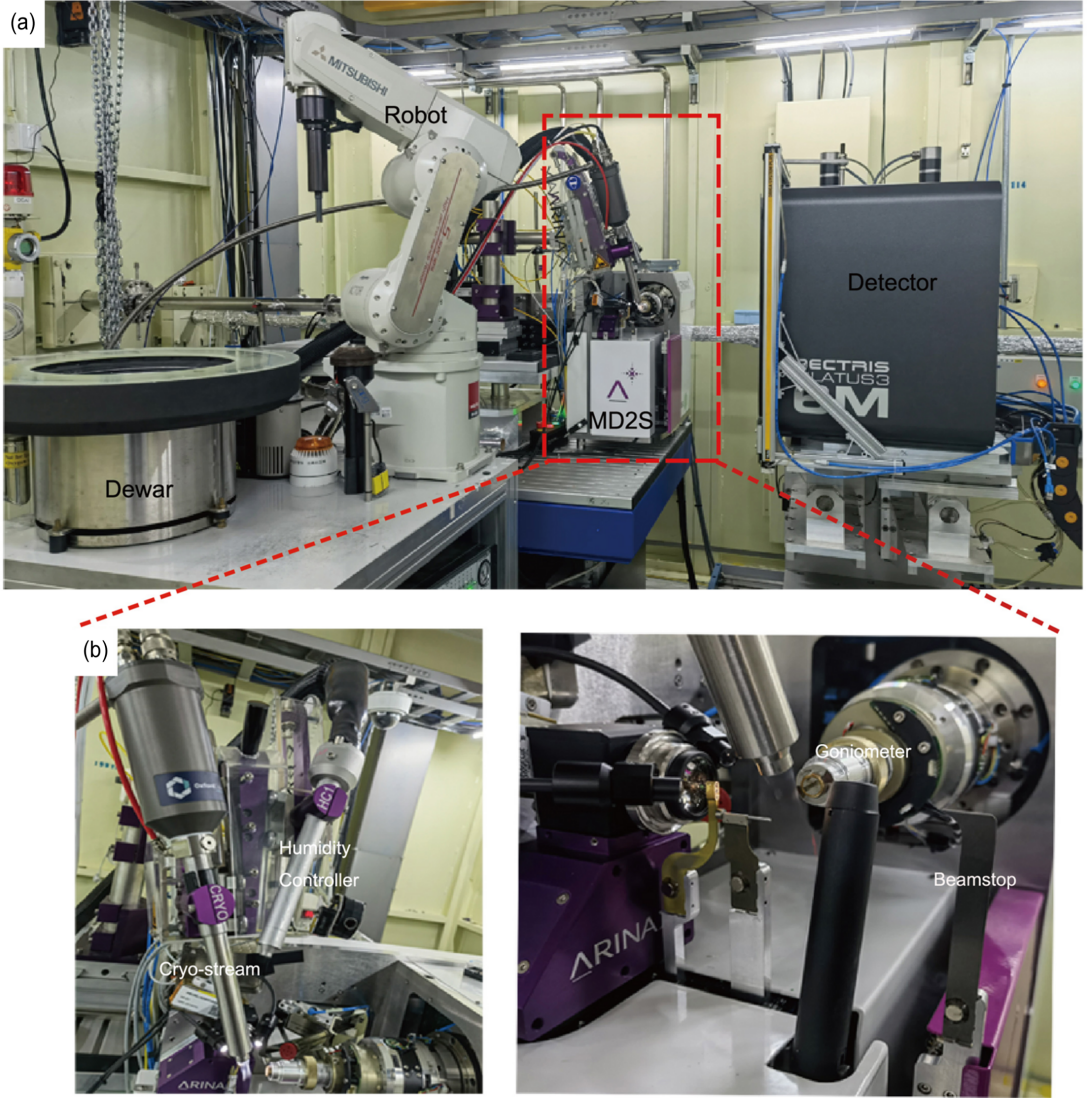
An overview of the enhanced experimental station on the BL19U1 beamline. (*a*) This part of the experimental station includes a robotic arm for sample transfer, a diffractometer for crystal alignment and data collection, and a detector for capturing diffraction patterns. (*b*) An enlarged view showing the HC that adjusts the microclimate for the crystals and the cryo-stream used to keep the crystals at a stable low temperature, and a section of the diffractometer for detailed sample positioning.

An HC with a rapid nozzle exchanger (REX) (https://www.arinax.com/hc-lab-humidity-controller/) has been integrated into the experimental platform, seamlessly interfacing with the cryo-stream within the same control panel. This integration enables researchers to switch effortlessly between different modes, tailoring the experimental conditions to their specific requirements. The detailed layout of these enhancements is illustrated in Fig. 2 ▸, showing the strategic positioning and interconnection of the upgraded components within the experimental station. These advances collectively position BL19U1 at the forefront of cutting-edge crystallography, providing researchers with state-of-the-art tools to unravel the intricacies of biological macromolecules and accelerate drug discovery processes.

The Fig. 2 ▸(*a*) provides a concise overview of the upgraded experimental station on the BL19U1 beamline, featuring key upgraded components such as the MD2S diffractometer, PILATUS3 6M detector and automatic sample-changer system. The red rectangular outline zooms in on the MD2S, the REX with cryo-stream and the HC. Fig. 2 ▸(*b*) highlights the integrated HC and the cryo-stream (left-hand panel) and the goniometer and movable beamstop (right-hand panel).

## 3. Diffractometer

**Figure 3:**
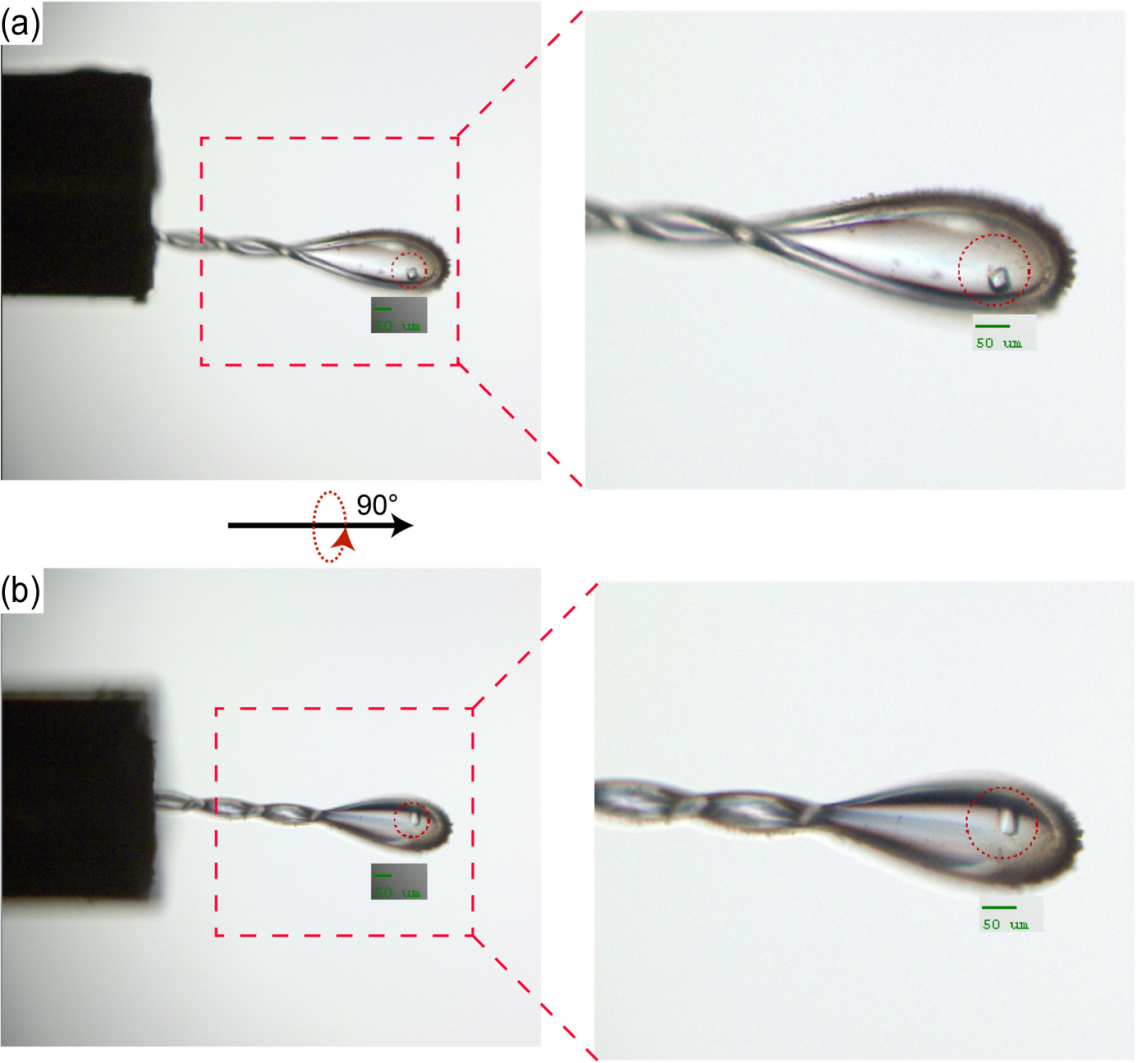
Sample alignment images from the BL19U1 experimental station. (*a*) A sample centred in the initial position. The image shows a crystal mounted on the goniometer pin, perfectly aligned within the red circle which indicates the focus area of the beam, as shown by the green scale bar indicating 50 µm. (*b*) The same sample after a 90° rotation. The crystal remains precisely centred, maintaining its position within the focus area, demonstrating the accuracy of the goniometer’s rotation and the stability of the sample mounting.

Ensuring precise sample alignment using the diffractometer is a pivotal step directly impacting user operations. In the evaluation of the performance and accuracy of the newly implemented diffractometer, our focus was on crystals with diameters less than 50 µm during experimental testing. The choice to test with such small crystals was based on the premise that these crystals present a more challenging scenario for alignment and visualization. Since small crystals require precise alignment for accurate data collection, they serve as an effective test of the diffractometer’s capabilities. Successfully aligning and collecting data from these smaller crystals assures us of the diffractometer’s performance with larger crystals, which most users typically work with. Furthermore, the improved visualization capabilities of the upgraded diffractometer are more evident when working with small crystals. This enhancement is particularly beneficial for accurately positioning and collecting data from crystals that are otherwise difficult to observe due to their size. Fig. 3 ▸(*a*) shows the position of one such small crystal after centring. The crystal is clearly within the red circle, *i.e.* this position aligns precisely with the location of the X-ray source. This precise alignment is indispensable for the experiment’s success.

In Fig. 3 ▸(*a*), right-hand panel, a magnified view of the crystal provides enhanced clarity for observing its precise positioning. This closer examination allows for a detailed scrutiny of the crystal’s alignment within the experimental setup. Fig. 3 ▸(*b*) depicts the altered position of the crystal following a 90° rotation of the goniometer. The crystal maintains its alignment with the optical path, remaining steady within the confines of the red circle. This controlled rotation demonstrates the stability and precision achieved by the new diffractometer.

These experimental results conclusively show that, even when dealing with crystals possessing a diameter of less than 50 µm, the newly implemented diffractometer consistently achieves highly accurate and stable alignment. This level of precision is imperative for the success of diffraction experiments, underscoring the efficacy of the upgraded equipment in catering to the intricacies of experimental requirements.

## 4. Humidity controller

The HC is a machine designed to control the relative humidity surrounding a sample in a crystallographic experiment. As its nozzle has the same dimension as a cryocooler it can be installed directly on a beamline. The HC device assumes a crucial role in crystal diffraction experiments conducted at room temperature as it precisely regulates the humidity surrounding the sample, ensuring that water molecules on the crystal surface do not evaporate. Consequently, the original shape and structure of the crystal are preserved, enabling researchers to conduct experiments at room temperature and circumventing potential complications arising from the freezing process.

**Figure 4:**
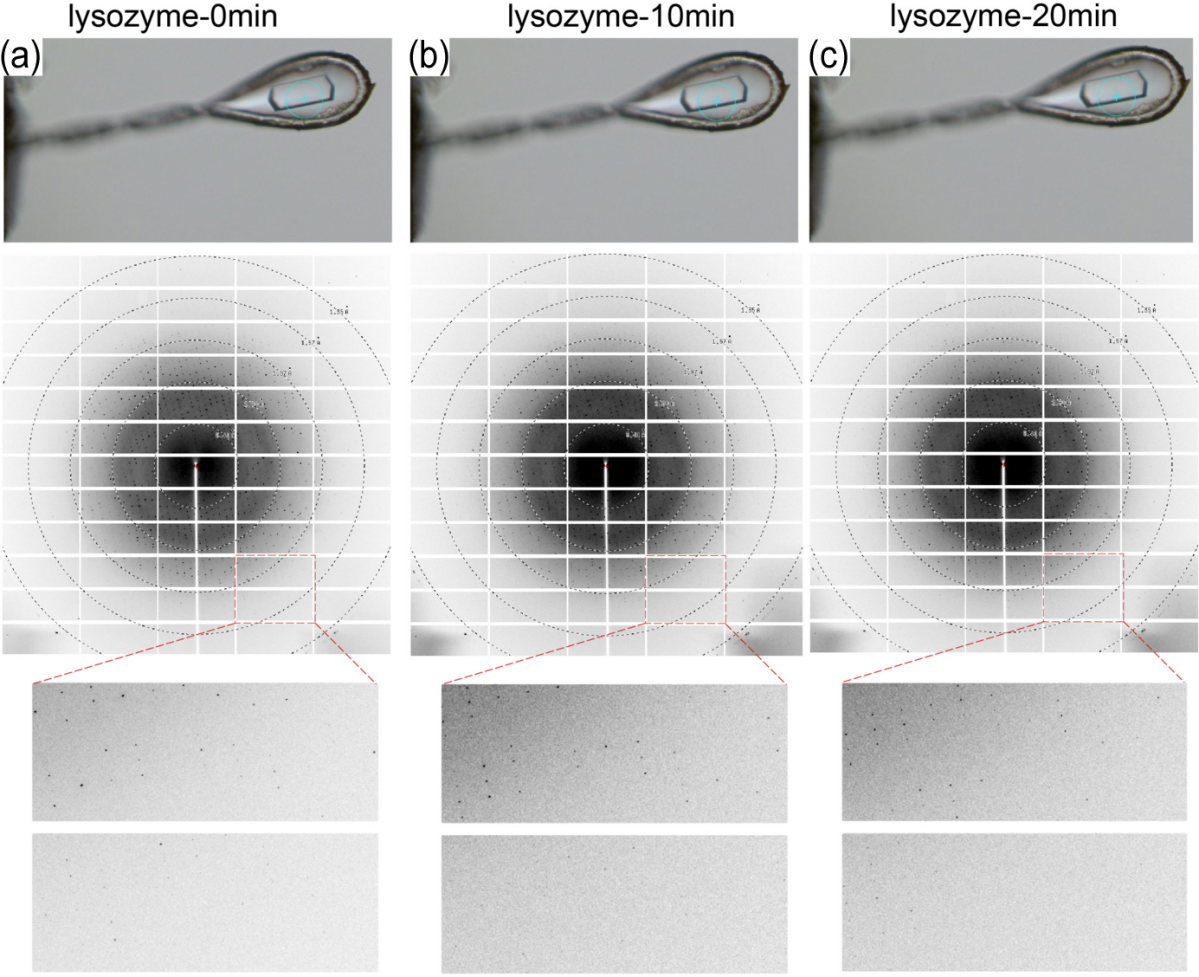
Sequential depictions of the impact of the HC on a lysozyme crystal during an experiment. (*a*) ‘lysozyme-0 min’ illustrates the crystal’s initial state and the corresponding diffraction pattern. (*b*) ‘lysozyme-10 min’ reflects the crystal’s condition 10 min after activating the HC. (*c*) ‘lysozyme-20 min’ captures the crystal’s state 20 min after activating the HC.

A crystal diffraction experiment was conducted under controlled conditions, specifically utilizing a humidity-controlled room at ambient temperature. The humidity source is double-distilled water, with a temperature of 20°C and humidity settings at 99% RH. Fig. 4 ▸ provides a visual representation of the crystal’s state and diffraction pattern at various time intervals during the utilization of the HC. Fig. 4 ▸(*a*) captures the crystal’s condition as the HC is activated. The diffraction pattern, measuring approximately 1.6 Å, reveals the crystal’s clear structure. Figs. 4 ▸(*b*) and 4 ▸(*c*) portray the crystal’s state after 10 and 20 min, respectively. Compared with the initial state, the crystal exhibits minimal changes, underscoring the HC’s effectiveness in preventing the evaporation of surface water molecules. The diffraction patterns in these later images closely resemble that in Fig. 4 ▸(*a*), indicating that the crystal’s diffraction capability remains largely unaffected during this timeframe.

In summary, these findings underscore the pivotal role of the HC in safeguarding the crystal’s state and preserving its exceptional diffraction capability. The demonstrated effectiveness of the HC validates its utility in facilitating diffraction experiments at room temperature, offering researchers a valuable tool for maintaining the integrity of crystal samples and obtaining reliable diffraction data.

## 5. New data collection system *MXCuBE3*

*MXCuBE3* (https://github.com/mxcube/mxcubeweb) stands out as a highly favoured crystal diffraction data collection system in current scientific endeavours. It not only shares functional similarities with the previous software used on BL19U1 (*Blu-Ice*; https://smb.slac.stanford.edu/facilities/software/blu-ice/getting_started.html) but also boasts distinct advantages that elevate its utility. Compared with *Blu-Ice*, *MXCuBE3* presents a more intuitive and modern user interface, simplifying operational procedures for users. Its compatibility with various communication protocols for diverse devices allows seamless integration of equipment, facilitating functional extensions (Gabadinho *et al.*, 2010 ▸).

**Figure 5:**
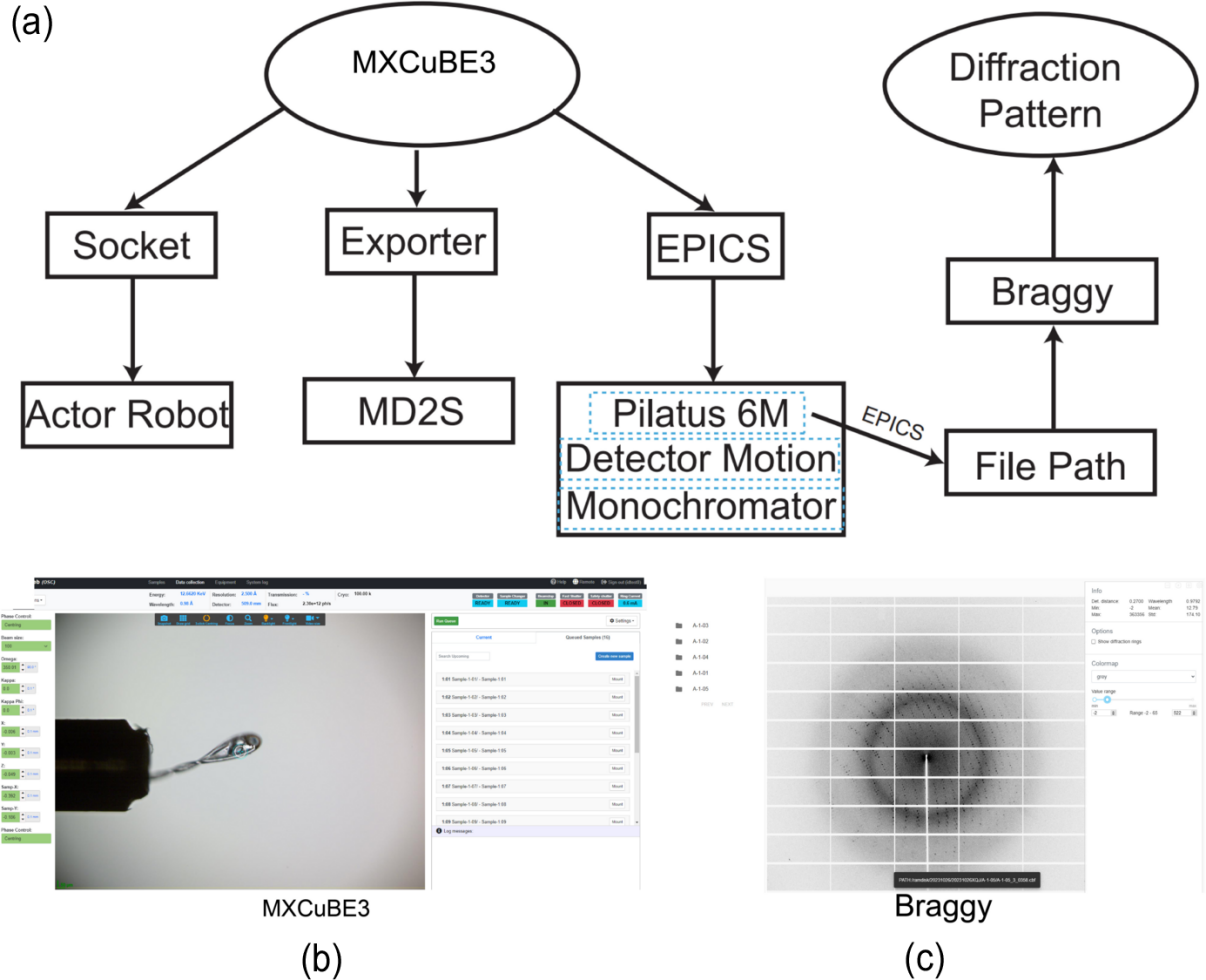
The integration and workflow of *MXCuBE3*. (*a*) A schematic representation of *MXCuBE3*’s interface and the communication protocols among various hardware components. (*b*) The *MXCuBE3* graphical user interface in action, showing a sample mounted on the goniometer ready for data collection. (*c*) The *Braggy* software interface displaying a diffraction pattern, illustrating the results obtained from an experiment conducted with the integrated system.

The widespread adoption of *MXCuBE3* is evident on a global scale, with numerous beamlines, including the BioMAX beamline at MAX IV, incorporating this system into their platforms for successful drug screening, yielding commendable results (Kozielski *et al.*, 2022 ▸; Lima *et al.*, 2020 ▸). The recent integration of the *MXCuBE3* system on BL19U1 further emphasizes its growing presence. The communication dynamics between *MXCuBE3* and the hardware control software, illustrated in Fig. 5 ▸, exemplify the system’s versatility.

Interfacing with the MD2S occurs through the *Exporter* protocol, providing direct control over various built-in MD2S functions in *MXCuBE3*. This includes managing the diffractometer status, automated sample alignment, employment of spiral data collection methods and so on. Meanwhile, communication with the ACTOR robotic arm (https://www.rigaku.com/products/crystallography/actor2) utilizes the *SOCKET* protocol, offering direct control over sample scratching within *MXCuBE3*. For the PILATUS 6M detector, both *EPICS* (https://epics.anl.gov/) and *Lima* (https://github.com/esrf-bliss/lima-camera-pilatus) are supported for communication. In practical applications, *EPICS* has demonstrated the more stable performance, leading to its selection for detector control, as depicted in Fig. 5 ▸(*a*).

*MXCuBE3* goes beyond conventional crystallography systems by supporting remote access via the internet. This feature enables users to collect data conveniently through a website interface. The system streamlines data collection by offering a pre-import feature for sample information, eliminating the need for users to enter details redundantly during experimentation. This not only saves time but also minimizes the potential for operational errors.

In the pursuit of enhancing the visualization of diffraction data, *MXCuBE3* employs *Braggy* (https://github.com/marcus-oscarsson/braggy) to observe diffraction patterns [Fig. 5 ▸(*b*)]. *Braggy*, akin to *adxv* (https://www.scripps.edu/tainer/arvai/adxv.html) and *ALBULA* (https://www.dectris.com/en/detectors/albula-software/) in function, stands out for its web-based viewing support, allowing researchers to access and share their diffraction data through a website [Fig. 5 ▸(*c*)]. This functionality significantly improves research convenience and efficiency. For real-time diffraction pattern display, *EPICS* is directly utilized to read the file path information collected by the detector and pass it to *Braggy*, creating a seamless and dynamic integration with the crystallography workflow. *MXCuBE3*, with its sophisticated features and integrative capabilities, continues to be a driving force in advancing crystallographic research methodologies.

## 6. Data collection

Diffraction data for a lysozyme crystal were collected utilizing the advanced *MXCuBE3* data collection system. The crystal conditions were carefully maintained at 0.1 *M* NaAc pH 4.6 (the pH was adjusted with citric acid), 18% NaCl and a temperature of 18°C. To ensure the crystal’s stability during data collection, a cryoprotective agent consisting of 20% glycerin was applied before subjecting it to liquid nitro­gen freezing.

Throughout the data collection stage, specific parameters were meticulously configured. The wavelength was set at 0.9793 Å, with a rotating angle of 0.5° per frame and an exposure time of 0.5 s. A total of 360 frames of data were collected systematically to capture a comprehensive data set. Following the completion of data collection, the *xia2–XDS* pipeline (Winter, 2010 ▸; Kabsch, 2010 ▸) was employed for subsequent processing and in-depth analysis of the acquired data.

**Figure 6:**
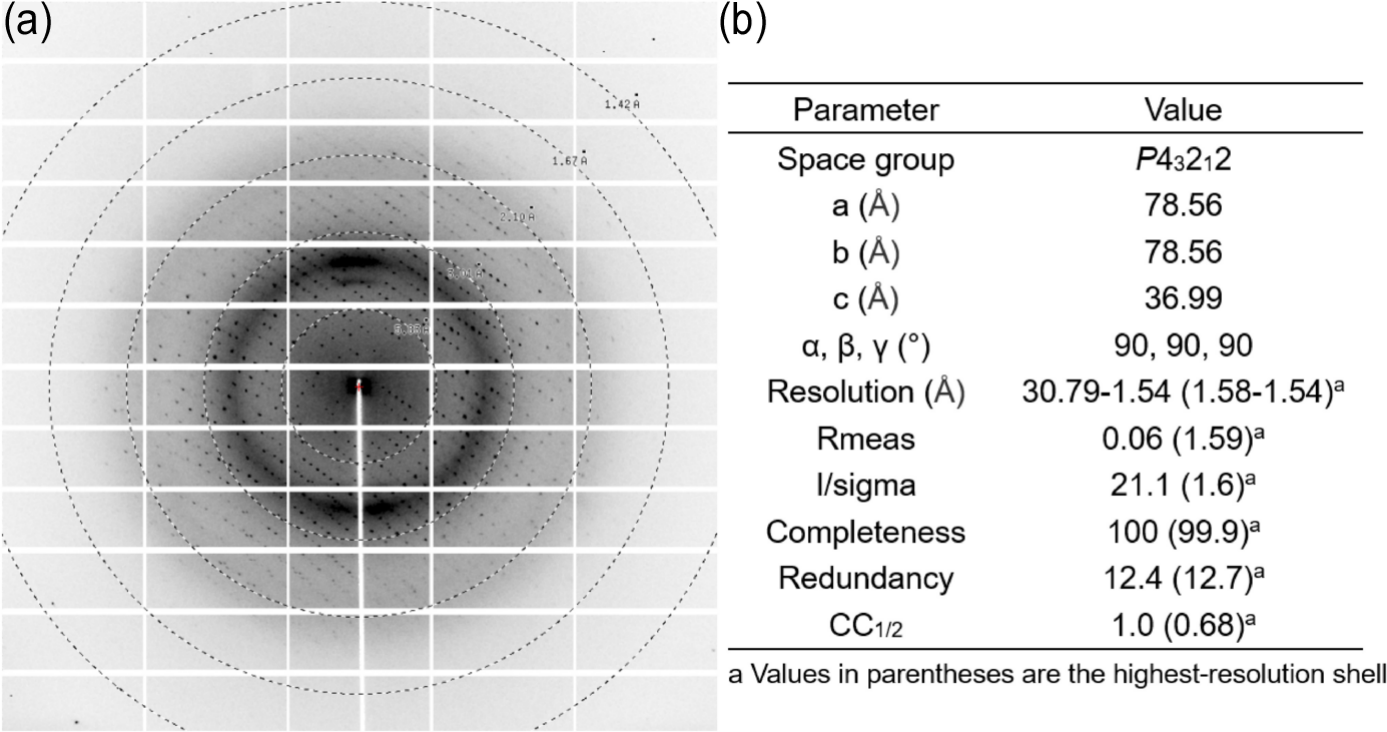
Data collection from a lysozyme crystal. (*a*) An X-ray diffraction pattern. (*b*) Statistical parameters.

A thorough examination of the X-ray diffraction pattern [Fig. 6 ▸(*a*)] reveals an impressive resolution, reaching as high as 1.6 Å. This outcome underscores the efficacy of the *MXCuBE3* system in delivering high-quality diffraction data. Analysis of statistical parameters [Fig. 6 ▸(*b*)] re­affirms that the upgrades made to the experimental station equipment have not compromised the robustness and reliability of the system but have successfully contributed to maintaining a high standard of data quality.

## 7. Diffraction data processing pipeline

With the aim of providing users with a streamlined method for promptly assessing the quality of diffraction data, formulating subsequent collection strategies and eliminating the need for manual data processing, we have implemented an automatic data processing pipeline. This integrated pipeline integrates the state-of-the-art diffraction data processing software tools *autoProc* (Vonrhein *et al.*, 2011 ▸ Kabsch, 2010 ▸), *xia2*–*XDS* (Winter, 2010 ▸; Kabsch, 2010 ▸) and *xia2*–*DIALS* (Winter, 2010 ▸, 2018 ▸). [*xia2*–*XDS* uses the xia2 pipeline=3d command line to tell *xia2* to use *XDS* and *XSCALE* to process data. *xia2*–*DIALS* uses the xia2 pipeline=dials command line to tell *xia2* to use *DIALS* to process data.] The primary objective of this pipeline is to offer users timely and automated processing results.

In order to establish an automated data processing pipeline, a local area network (LAN) was set up for efficient data transfer. The storage folder, boasting a 24 TB capacity, is located on the PPU1 computer and is directly mounted to the detector computer. This arrangement allows for the immediate collection of data from the detector into the PPU1 storage folder. Once data collection is complete, the data are then transferred to a 9.8 TB capacity storage folder on the PPU2 computer. The data processing pipeline is configured to access and process diffraction data from this storage on PPU2, ensuring a streamlined and efficient workflow for handling large volumes of crystallographic data. The data are also transferred from PPU2 to a data storage unit with 216 TB capacity as a cold backup.

**Figure 7:**
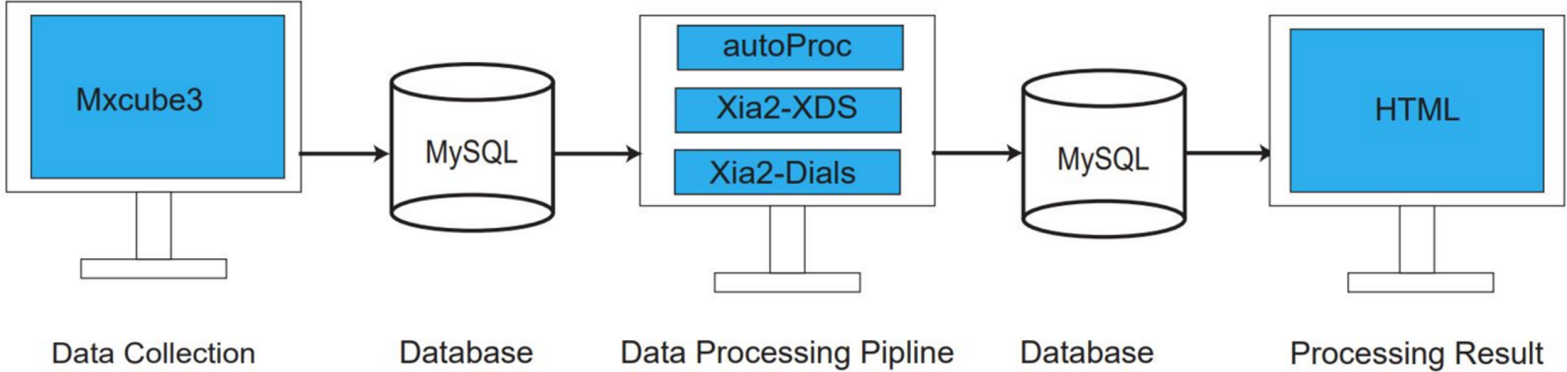
The automated data processing workflow pipeline.

The workflow of the pipeline is structured as depicted in Fig. 7 ▸. As users collect data through *MXCuBE3*, essential parameters such as file paths, the number of collected images and information about the success of data collection are recorded and stored in a MySQL database (https://www.mysql.com/). Using this stored information, the data processing pipeline then automatically processes the collected diffraction data. Upon completion of the processing phase, the results are saved to another MySQL database and presented as a user-friendly webpage (HTML format). The diagram provides a clear visualization of this automated data processing workflow.

For users who may prefer or need to process diffraction data manually, our system also accommodates their preferences. We offer the *HKL3000* (Minor *et al.*, 2006 ▸) and *AUTOPX* (Wang *et al.*, 2022 ▸) software tools, providing users with convenient and swift options for manually processing data. This dual approach caters to the diverse needs of researchers, ensuring a seamless and efficient experience in handling diffraction data.

## 8. Facility access

The BL19U1 beamline, in alignment with the operational schedule of the SSRF, allocates approximately 3600 h of beam time annually to facilitate scientific research endeavours. The process for users involves submitting proposals in advance, and upon successful approval they obtain the necessary beam time to schedule their experiments. For academic research users, four types of proposal are accommodated: regular projects, key projects, award projects and urgent projects.

Regular projects, representing the primary avenue, offer two application opportunities each year, accounting for roughly 75% of the total beam time. Application deadlines for general projects fall on 30 March and 30 September, and the submission process is facilitated through the sharing service platform of the Chinese Academy of Sciences Larger Researcher Infrastructure (https://lssf.cas.cn). Beamline staff conduct a technical review to assess project feasibility, which is followed by peer review by committee members evaluating scientific merit. Beam time allocation is then based on the received scores.

In instances where users miss the regular proposal application windows, or in cases of urgent experimental demands, individuals can apply for urgent projects. These projects typically do not exceed the average user hours of the previous quarter. Key projects offer an annual application opportunity, providing researchers with additional hours to conduct comprehensive experiments.

Outstanding contributors can submit their achievements via the feedback system to be considered for the award project category, comprising no more than 15% of the annual beam time. Allocation criteria for this category are contingent on the quantity of applications and the likely impact of the outcomes.

For industrial users, an annual allocation of approximately 10% of the beam time is earmarked, with this percentage progressively increasing. Industrial users can initiate the application process through the user office.

Once approved, the allocated beam time remains valid for two years. To ensure safety compliance, users are required to undergo online safety training and successfully pass a safety examination. Scheduling beam time at least one month in advance is a prerequisite for accessing BL19U1, and during experiments, BL19U1 staff provide real-time technical support to ensure the smooth progression of each user’s research endeavours.

## 9. Discussion and conclusions

In the realm of user services, BL19U1 has garnered noteworthy achievements in both PDB data releases and the publication of related articles. Since its initiation for user access, the beamline has facilitated the deposition of over 2000 PDB structures and the publication of more than 1000 scientific papers, including over 45 papers in prestigious journals such as *Nature*, *Science* and *Cell*. This robust track record undeniably attests to BL19U1’s outstanding performance in supporting scientific research endeavours.

However, recognizing that the exponential growth of data alone may not suffice to meet the evolving needs of increasingly complex research, BL19U1 has strategically prioritized the upgrade, modification and optimization of its crystallography experimental station’s software and hardware components. Following the successful completion of upgrades to the diffractometer, the installation of a new data collection system and the update of the automatic diffraction data processing pipeline, the next phase involves the upgrade of the Dewar and robot arm, and the configuration of the *ISPyB* database (https://github.com/ispyb/ISPyB).

These advances aim to enhance significantly the available sample loading capacity, loading efficiency and data management capabilities, aligning with the requirements of crystal fragment library based research. The envisioned transformation is substantial, with the system’s sample loading capacity projected to increase from 80 to over 400 crystals, accompanied by a significant reduction in sample loading speed from 1 min to within 20 s. Such enhancements will give researchers considerable time savings and heightened work efficiency during their experiments.

The overarching goal is to achieve a state of fully automated data collection, processing, structure analysis, automatic small-molecule matching and streamlined data management processes. In essence, BL19U1 aspires to be at the forefront of providing cutting-edge and efficient services for the scientific research community.

Despite the substantial contributions made in the past few years, the beamline’s potential for future growth remains significant. Continuous hardware and software system upgrades and optimizations are anticipated to strengthen BL19U1’s capacity to cater to the evolving needs of the scientific research community, ensuring it remains at the forefront of facilitating groundbreaking research.
